# Constructing indicator species distribution models to study the potential invasion risk of invasive plants: A case of the invasion of *Parthenium hysterophorus* in China

**DOI:** 10.1002/ece3.10672

**Published:** 2023-11-01

**Authors:** Jiamin Liu, Haiyan Wei, Jiaying Zheng, Ruidun Chen, Lukun Wang, Fan Jiang, Wei Gu

**Affiliations:** ^1^ National Engineering Laboratory for Resource Development of Endangered Crude Drugs in Northwest China Shaanxi Normal University Xi'an China; ^2^ School of Geography and Tourism Shaanxi Normal University Xi'an China; ^3^ College of Life Sciences Shaanxi Normal University Xi'an China

**Keywords:** ensemble model, generalized joint attribute model, indicator species, indicator species distribution models, invasive plants, potential invasion risk, spread

## Abstract

**Aim:**

As invasive plants are often in a non‐equilibrium expansion state, traditional species distribution models (SDMs) are likely underestimating their suitable habitat. New methods are necessary to identify potential invasion risk areas.

**Location:**

Tropical monsoon rainforest and subtropical evergreen broad‐leaved forest regions in China.

**Methods:**

We took *Parthenium hysterophorus* as a case study to predict its potential invasion risk using climate, terrain, and human activity variables. First, a generalized joint attribute model (GJAM) was constructed using the occurrence of *P. hysterophorus* and its 27 closely related species in Taiwan, given it is widely distributed in Taiwan. Based on the output correlation values, two positively correlated species (*Cardiospermum halicacabum* and *Portulaca oleracea*) and one negatively correlated species (*Crassocephalum crepidioides*) were selected as indicator species. Second, the distributions of *P. hysterophorus* and its indicator species in the study area were predicted separately using an ensemble model (EM). Third, when selecting indicator species to construct indicator SDMs, two treatments (indicator species with positive correlation only, or both positive and negative correlation) were considered. The indicator species' EM predictions were overlaid using a weighted average method, and a better indicator SDMs prediction result was selected by comparison. Finally, the EM prediction result of *P. hysterophorus* was used to optimize the indicator SDMs result by a maximum overlay.

**Results:**

The optimized indicator SDMs prediction showed an expanded range beyond the current geographic range compared to EM and the thresholds for predicting key environmental variables were wider. It also reinforced the human activities' influence on the potential distribution of *P. hysterophorus*.

**Main Conclusions:**

For invasive plants with expanding ranges, information about indicator species distribution can be borrowed as a barometer for areas not currently invaded. The optimized indicator SDMs allow for more efficient potential invasion risk prediction. On this basis, invasive plants can be prevented earlier.

## Graphical abstract

Joint species distribution models (JSDMs) can be used to select indicator species for invasive plants. Optimized indicator SDMs can facilitate large‐scale studies and predict a wider range of spatial and environmental niches.

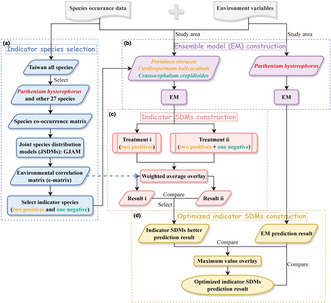

## 1 INTRODUCTION

Biological invasion has been widely recognized as a major ecological problem at the global and local levels (Brown et al., 2020; Zedda et al., 2010). Invasive plants affect native individuals, genetic effects, population dynamic effects, community effects, ecosystem process, and services (Bartz & Kowarik, 2019; Kueffer, 2017; Parker et al., 1999) and cause significant economic losses worldwide (Gren et al., 2009; Liu et al., 2010; Marbuah et al., 2014). Because of the potential adverse impacts of invasive species, it is valuable for conservation biologists as well as practitioners how to effectively manage them and accurately predict geographic areas at invasion risk (Mothes et al., 2019).

How to monitor the expansion trends of invasive species and reduce negative environmental impacts often relies on habitat suitability modeling and decision analysis (Tonini et al., 2017). Species distribution models (SDMs) use species occurrence sites and associated environmental data to map species niches in geographic and environmental space (Elith & Leathwick, 2009). SDMs are therefore increasingly used to predict the distribution of invasive species and understand the drivers of biological invasions (Elith et al., 2011; Fang et al., 2021; Zhang et al., 2020). The ensemble model (EM) is considered one of the most promising approaches in SDMs because it avoids over‐reliance on one particular model by averaging the multiple models' predictions (Araújo & New, 2007). EM has been proven to improve model predictions and reduce overfitting (Breiner et al., 2015; Oppel et al., 2012; Stohlgren et al., 2010).

A key assumption in SDMs is that species are in equilibrium with their environment in the area used to train the model (Elith et al., 2010; Journé et al., 2020; Srivastava et al., 2021). However, invasive species are usually in disequilibrium with their environment, especially in the early stages of invasion and establishment, e.g., not have enough time to fully occupy all suitable environmental ecological niches or temporarily occupy unsuitable habitats (Elith et al., 2010; Hattab et al., 2017; Uden et al., 2015). Besides, invasive species may have expanded their realized niche in the invaded area, in which case the predicted range will be inaccurate (Srivastava et al., 2021). In recent years, several studies have demonstrated the limitations of SDMs in predicting invasive species distributions at new geographic ranges or time periods (Liu et al., 2020; Yates et al., 2018). To improve the models' predictive power, we need to understand how invasive species respond to new environments (Merow et al., 2017). However, these require field experiments and are difficult to generalize to large numbers of species. Therefore, we need to use additional information beyond the current potential distribution range to predict the distribution of invasive species (Gallien et al., 2012; Pili et al., 2020).

Many studies have begun to model the distribution of study species by borrowing information on the distribution of other related species. Most previous studies relied on highly dependent relationships between species when selecting related species, such as parasitic relationships (Araújo & Luoto, 2007; Schweiger et al., 2012; Singer et al., 2018) and trophic relationships (Mäkinen & Vanhatalo, 2018). The distribution status or potential distribution of dominant or highly related species such as cover and density is often used as a predictor in studies (Mod et al., 2015; Pellissier et al., 2010). In other studies, the environmental responses of all species are first clustered, and multiple species with similar environmental responses to the study species are selected. Combined response information from multiple species is then used to correct the distribution of the studied species (Hui et al., 2013; Larson et al., 2014). This approach relies on the investigation of communities and cannot be extended to large‐scale spaces. Referring to the indicator species concept (Landres et al., 1988), species that have similar environmental responses to invasive species were named indicator species in this article. And use the distribution information of indicator species to conduct reasonable monitoring of areas that are initially invaded or have not yet been occupied.

How to select indicator species for invasive species and use their distribution information rationally is an open question (Larson et al., 2014). In this article, we chose joint species distribution models (JSDMs) to select indicator species. JSDMs can establish species responses to multiple environmental variables and capture associations among species in the form of random effects (Harris, 2015; Warton et al., 2015). JSDMs are powerful tools for analyzing interspecific relationships, but they are not suitable for predicting the distribution of individual species, especially for rare and endangered species, as they may not directly predict the distribution of invasive species (Norberg et al., 2019; Zhang et al., 2018; Zurell et al., 2018). The generalized joint attribute model (GJAM) is an extension of JSDMs that enables the modeling of multiple types of species observations. It can explicitly calculate species' environmental response correlations on the scale of input data, providing new inferences and predictions for the flexibility of ecological data (Clark et al., 2017). Hence, based on its output of environmental correlation information, the indicator species corresponding to the invasive species can be identified. Meanwhile, indicator species should be the most widely distributed native or endemic that are more likely to be in equilibrium and more consistent with the modeling assumptions.

*Parthenium hysterophorus* L. is an annual herb of the Asteraceae family, native to Texas and northern Mexico (Navie, 1996), and is considered one of the world's most invasive weeds (Bajwa et al., 2017). In China, specimens were first collected in Yunnan (1926) and have invaded parts of south China (Zhu et al., 2005). This plant reproduces mainly by seeds and it usually produces numerous seeds (Dhileepan, 2012), with a high survival and germination rate under appropriate conditions (Gnanavel & Natarajan, 2013; Nguyen et al., 2017). *P. hysterophorus* is adapted to a wider range of climatic conditions and can grow on a variety of soil types (Evans, 1987). *P. hysterophorus* also produces allelopathic chemicals that inhibit the growth of adjacent vegetation, and these factors contribute to the vigorous growth and rapid spread of *P. hysterophorus* (Belgeri et al., 2012). The impact of *P. hysterophorus* is multifaceted and its invasion has caused serious damage in many countries, resulting in significant economic and ecological losses (Navie, 1996; Nyasembe et al., 2015). Several studies have been conducted using traditional SDMs to predict the distribution of *P. hysterophorus* (Al Ruheili et al., 2022; Maharjan et al., 2019; Shrestha et al., 2015).

In this study, *P. hysterophorus* was used as a case study to predict the potential invasion risk by borrowing information from the distribution of indicator species. Our aims are (a) to select the appropriate indicator species for *P. hysterophorus*; (b) to use the indicator SDMs to identify environments that are suitable for *P. hysterophorus* but not currently occupied; (c) to predict the potential invasion risk of *P. hysterophorus* by optimized indicator SDMs; (d) to analyze the reasons for the expanded invasion risk range of *P. hysterophorus* by comparing the EM and optimized indicator SDMs predictions, and make reasonable *P. hysterophorus* management recommendations for different situations of invasion risk.

## 2 METHODS

### 2.1 Study area

**FIGURE 1:**
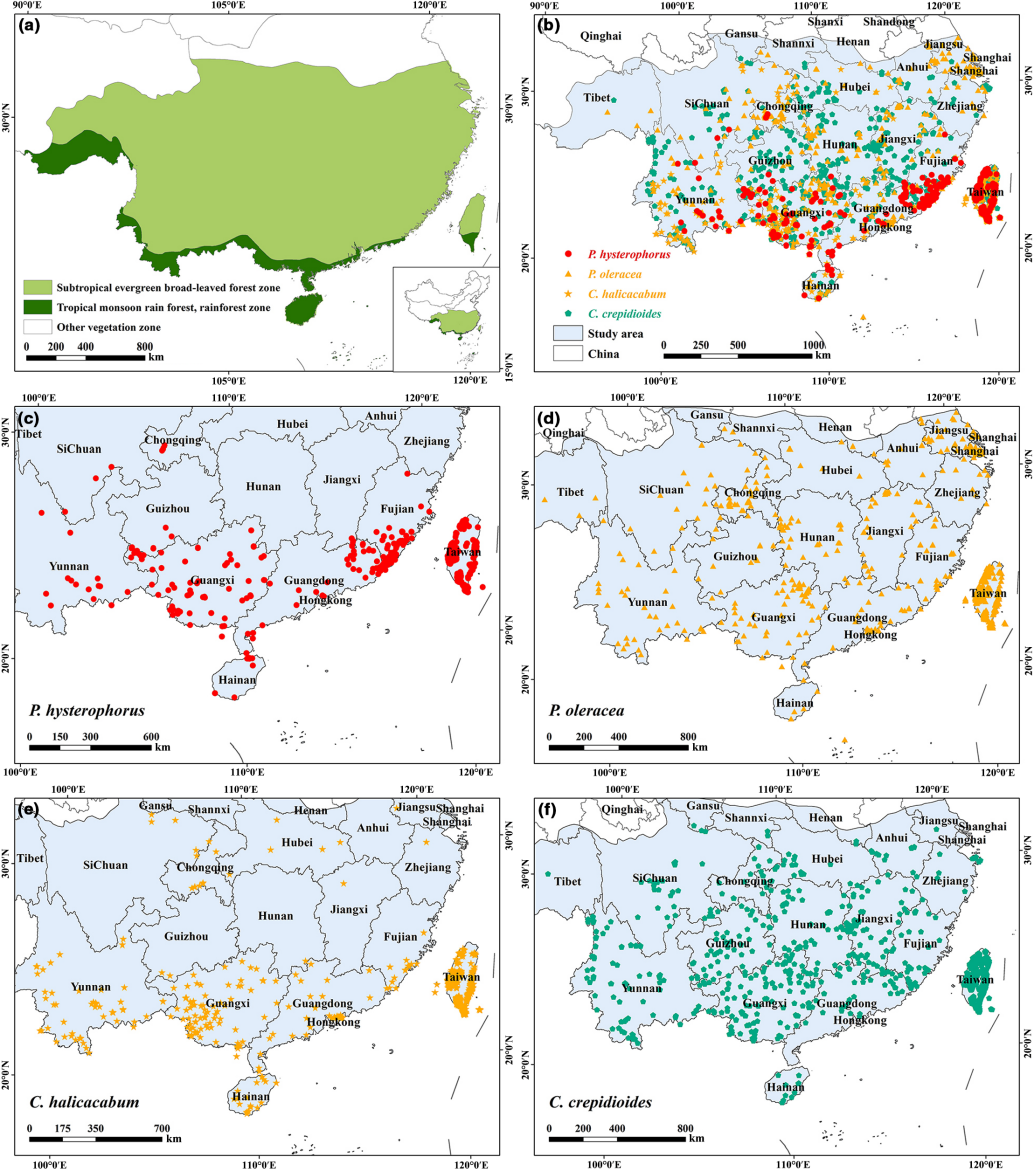
Study area and sampling locations of species in China. (a) The study area of this article, the dark green region, and the light green region are subtropical evergreen broad‐leaved forest zone and tropical monsoon rain forest, rainforest zone, respectively. (b) The light blue area is the study area. The red dots represent the invasive plant *Parthenium hysterophorus*. The yellow dots are those with a positive correlation with *P. hysterophorus*, and the green dots are those with a negative correlation. (c) *P. hysterophorus* (428 points). (d) *Portulaca oleracea* (693 points). (e) *Cardiospermum halicacabum* (760 points). (f) *Crassocephalum crepidioides* (1236 points).

The scope of this study is the tropical monsoon rainforest region and subtropical evergreen broad‐leaved forest region in China (Figure 1a). Vegetation zoning data were obtained from Peking University (http://geodata.pku.edu.cn). With strong solar radiation and abundant hydrothermal conditions, the study area is not only a hotspot of biodiversity in the world but also an area with a high degree of plant diversity and specificity (Ye et al., 2017). Simultaneously, it is also seriously threatened by biological invasion (Wang et al., 2017).

### 2.2 Species occurrence record data

The species occurrence data used in this study were obtained from the Global Biodiversity Information Platform (GBIF, http://www.gbif.org; Table S1), the Chinese Virtual Herbarium (http://www.cvh.ac.cn), and the National Specimen Information Infrastructure (http://www.nsii.org.cn). To better simulate the actual distribution and reduce spatial bias, for all species occurrence data used in this study, we removed duplicated records within the area of 1 km spatial resolution, inaccuracies (<0.1 decimal degrees), and coordinates beyond the study's boundary.

In mainland China, *P. hysterophorus* was probably first transferred from Vietnam to Yunnan Province in southern China in 1926 but did not begin to expand further until the late 1980s (Towers & Mitchell, 1983). In recent years, it tends to have spread further northwards (Chen et al., 2016; Lu et al., 2020). On the island of Taiwan, it was first recorded in 1986 in Kaohsiung (Peng et al., 1988), since then, the weed has spread northwards; it now has populations all over the island (Mao et al., 2021). *P. hysterophorus* is more widely distributed on the island of Taiwan than in mainland China (Figure 1c), and the species is more likely to be in relative equilibrium. According to Köppen‐Geiger climate classification maps (Beck et al., 2018), the continental part of the study area is consistent with the climate zoning of Taiwan. Taiwan, as a continental island, once belonged to a unified flora with mainland China (Chen et al., 2012). Several studies have shown that there is a continuous distribution of taxa and great similarity in flora between mainland China and Taiwan (Chen et al., 2012; Wen et al., 2020; Zhu, 2016). In addition to these reasons, Taiwan has rich topographic conditions with plains, basins, tablelands, hills, and mountains. GJAM is unsuitable for large‐scale data, and Taiwan, with its small size and wide coverage of the environment, is the relatively most suitable area, so we used species occurrence data from Taiwan to construct GJAM to identify environmental correlations between species. Data from the entire study area were used to model species distributions of individual species.

### 2.3 Environmental variables and selections

Climate is the main driver of species distribution (Pearson & Dawson, 2003; Ranjitkar et al., 2016). In addition, other environmental factors may also influence species distribution (Austin & Van Niel, 2011; Bucklin et al., 2015; Heikkinen et al., 2006), such as soil variables can contribute to the habitat suitability of plant species at macroecological scales (Figueiredo et al., 2018); terrain can influence climate, soil, seed migration, and development of roots (Youcefi et al., 2020); human activities are drivers of global plant invasions and influence the range of plants in human‐disturbed environments (Beans et al., 2012; Mairal et al., 2022). Considering that *P. hysterophorus* can be adapted to different soil environments (Evans, 1987), climatic variables, terrain variables, and human activity variables were considered to construct the model. Details of the selected environment variables are in Table S2.

In species distribution modeling, overparameterized models with too many predictor variables can fit complex relationships in the training domain but are likely to produce unreliable predictions (Petitpierre et al., 2017). Besides, as more variables are included in the model, its prediction range becomes narrower (Beaumont et al., 2005; Braunisch et al., 2013). Therefore, we performed the following operations to select the minimum number of feasible predictors. First, the co‐linearity between variables can lead to overfitting of the model and affect the prediction results, so we calculated Pearson correlation coefficients between variables and retained the meaningful variables with |*r*| < .8 (Bucklin et al., 2015; Wan et al., 2017; Zhao et al., 2021). Additionally, in JSDMs, since all species share a common set of predictor variables, we performed principal component analysis (PCA) on the variables to identify a subset that best captures all dimensions of environmental variation (König et al., 2021). Variables were ranked according to their loadings on each principal component, and the variable with the highest loading was selected. Also considering species habitat (Table S3), seven variables were finally retained, namely Bio1, Bio4, Bio12, Bio15, elevation above sea level (EASL), aspect (ASPE), and human footprint (HFP) (Table 1, Figure S1). The four screened climate variables have been shown to influence the distribution of plant species at different spatial scales and have been widely used to predict the distribution of suitable habitats for plant species (Kelly et al., 2014; Wan et al., 2014; Wang & Wan, 2021).

**TABLE 1:** Environmental variables used in GJAM and SDMs.

Environment variables	Description	Abbreviation	Unit
Climate	Annual mean temperature	Bio1	°C
	Temperature seasonality (standard deviation × 100)	Bio4	–
	Annual precipitation	Bio12	mm
	Precipitation seasonality (Coefficient of variation)	Bio15	–
Terrain	Elevation above sea level	EASL	m
	Aspect	ASPE	°
Human activity	Human footprint dataset	HFP	–

### 2.4 Selection indicator species using GJAM

We used the R package “gjam” to construct the GJAM (Clark et al., 2017). GJAM construction requires three elements, two of which are environmental variables and species attributes, collectively called the observations, $\left\{x_{i},y_{i}\right\},i=1,\ldots ,n$. The vector $x_{i}$ contains predictors $x_{\mathrm{iq}}:q=1,\ldots ,Q$. The vector $y_{i}$ contains attributes (responses), such as species abundance, presence‐absence (PA), and so forth, $y_{\text{is}}:s=1,\ldots ,S$. The remaining element is sampling effort $E_{\text{is}}$, which invested to obtain the observation of response at a location can affect the observation.

**FIGURE 2:**
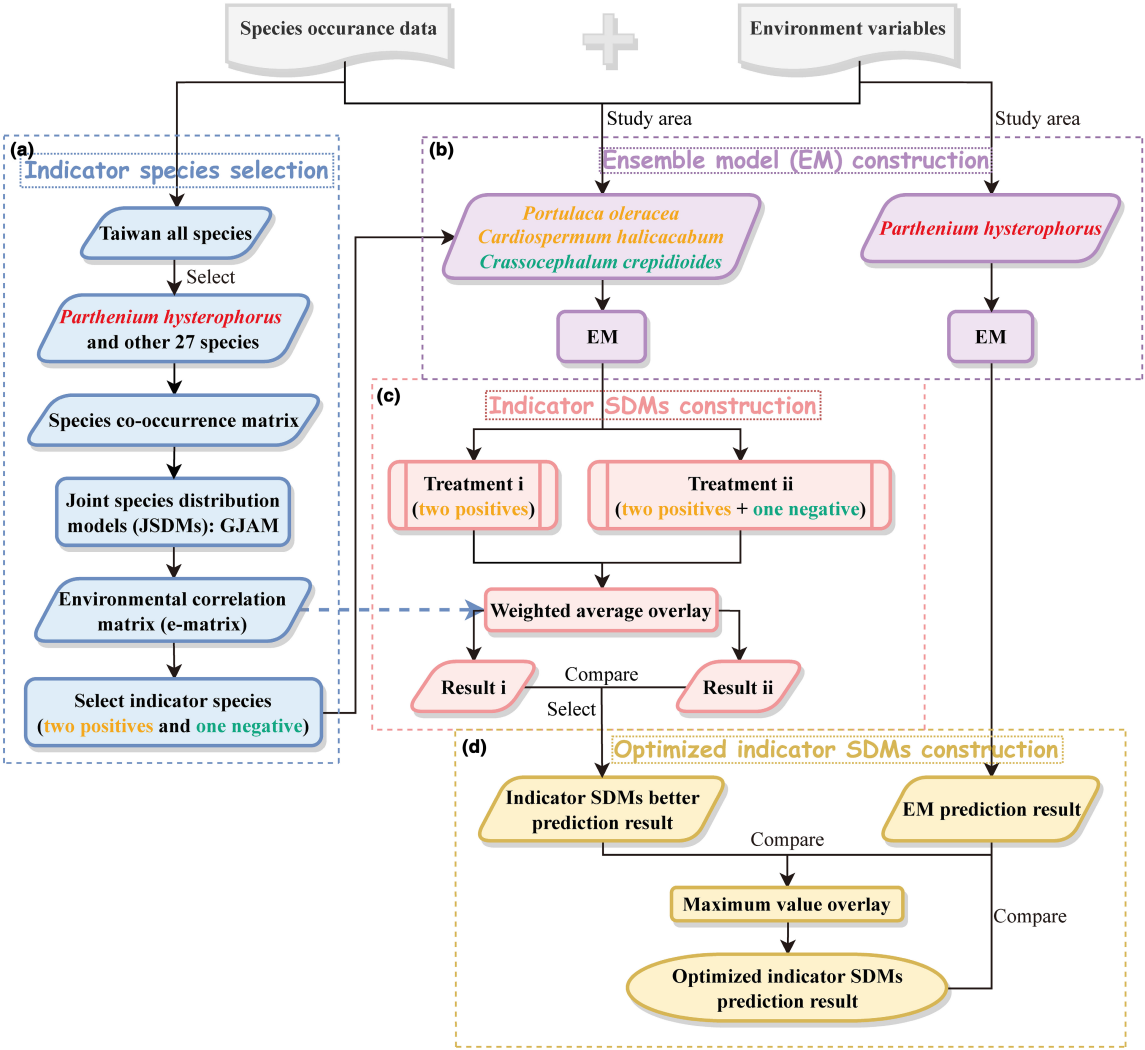
Indicator SDMs construction flowchart. (a) The process for selecting indicator species using GJAM. (b) The process of using EM to predict the distribution of *Parthenium hysterophorus*, and the three indicator species. (c) The process of constructing indicator SDMs. (d) The process of constructing optimized indicator SDMs.

For the observation data, we created a species co‐occurrence matrix using the data from Taiwan (Figure 2a). We first excluded rarely distributed species (less than 50 records). Second, species with few records throughout the study area were excluded. Third, species with more than 50% of the points were located within the 5 km buffer zone of *P. hysterophorus* occurrence data were selected. Ultimately, referring to a study on the *P. hysterophorus* community (Zhang et al., 2010), 27 community members were selected (Table S3, Figure S2), and a total of 16,837 presence points were used to construct the species co‐occurrence matrix.

We used the seven environment variables filtered above as predictors, which were Z‐Score normalized to calculate stability. As the occurrence sites were previously sampled at 1 km resolution, we assumed that the retained data points were all sampled perfectly uniformly, so equalized the effort to 1 at all locations. The “gjam” model was run for 100,000 generations with 1500 generations of burn‐in. For all variables, the Markov chain Monte Carlo (MCMC) convergence was visually checked (Figure S3). GJAM can use these input data to calculate the correlation matrix of the species' environmental response (e‐matrix), as well as the residual correlations (r‐matrix) between species that cannot be explained by environmental variables, such as species interactions, stochastic processes, etc. (Norberg et al., 2019). In this article, only the e‐matrix is considered to identify indicator species, retaining the most and least environmentally similar species.

### 2.5 EM construction and evaluation

In this study, we chose the EM to predict the distribution of individual species in the study area. The “biomod2” package (includes 11 different single models, Table S4) in the R platform (Thuiller, 2003; Thuiller et al., 2009) was used to model the geographic distribution of the invasive species and its indicator species. We invoked all models in the package. Besides, three pseudo‐absence data sets were randomly generated with an approximately equal number of points present (Barbet‐Massin et al., 2012). Then, 75% of the data samples were randomly selected as training data, and 25% of the samples were set as test data (Guo et al., 2016). For each species, the division of training and test data was repeated 10 times to obtain a total of 330 single models (11 kinds of models × 10 repetitions × 3 sets of sampled (presence and pseudo‐absence) data). Since the area under the relative operating characteristic (ROC) curve (AUC) values were all higher than the true skill statistic (TSS) values (See the details below), we chose the TSS values to filter the single models used to build EM. Based on the accuracy of each model for different species, we selected appropriate evaluation metric thresholds.

The weighted average method was used to construct the EM, i.e., the TSS values were used to calculate the weights of the filtered individual models of the EM. The weights were calculated by Equation (1).

(1) $$w_{i}=\frac{{\mathrm{TSS}}_{i}}{\sum_{i=1}^{n}{\mathrm{TSS}}_{i}}$$

$w_{i}$ denotes the weight of a single model, ${\mathrm{TSS}}_{i}$ is the value of *i*th model, and *n* is the total number of single models with TSS greater than the threshold value.

The habitat suitability index (HSI, threshold 0 ~ 1) calculated from SDMs is commonly used in research to describe the distribution of suitable habitats for species (Wang et al., 2021; Zhao et al., 2020). Based on this concept, for invasive species, the invasion risk index (IRI) was used to describe their invasion probability. We obtained the IRI of *P. hysterophorus* by EM, calculated as Equation (2).

(2) $${\mathrm{IRI}}_{\mathrm{EM}}=\sum_{i=1}^{n}\left(\sum_{j=1}^{m}w_{i}\times v_{\mathrm{ij}}\right)$$

${\mathrm{IRI}}_{\mathrm{EM}}$ is the IRI calculated by the EM, $v_{\mathrm{ij}}$ is the value of the *j*th raster cell in the *i*th single model, and *m* is the total number of raster cells of a single model result. The “biomod2” package contains five ways (“PA_dataset+repet”, “PA_dataset+algo”, “PA_dataset”, “algo” and “all”) of combining models to build EM, and we chose the “PA_dataset + repet” method, i.e., combining different groups of pseudo‐absence datasets and cross‐validation datasets.

Regarding previous studies (Fang et al., 2021; Wang et al., 2021), we reclassified IRI into four classes, with 0.7 < IRI ≤ 1 as high invasion risk, 0.5 < IRI ≤ 0.7 as moderate invasion risk, and 0.3 < IRI ≤ 0.5 as low invasion risk, and 0 ≤ IRI ≤ 0.3 as unsuitable. Finally, we simulated the distribution of *P. hysterophorus* and its indicator species using EM (Figure 2b).

We used the TSS (Allouche et al., 2006) and AUC (Swets, 1988) as indexes to assess model accuracy. The AUC is a graphical method defined by the sensitivity on the *Y*‐axis and the false‐positive ratio (1‐specificity) on the *X*‐axis (Fielding & Bell, 1997; Kwon et al., 2015). AUC is independent of prevalence and is considered to be an accurate measure of model performance (McPherson et al., 2004). The TSS (equal to $\text{sensitivity}+\left(\text{specificity}-1\right)$) takes into account the missing mean error and is not affected by the size of the validation set (Allouche et al., 2006). The higher the value of these two indexes, the higher the model prediction accuracy (Hipólito et al., 2015). Generally, when the TSS is greater than 0.8 and the AUC is greater than 0.9, the model fitting accuracy is very high (Zhang et al., 2020).

### 2.6 Indicator SDMs construction

To better predict the potential invasion risk of *P. hysterophorus*, we borrowed the distribution of indicator species to indicate the spread trend of *P. hysterophorus*. For this purpose, we constructed indicator SDMs and considered two treatments: (i) use only the species positively correlated with the *P. hysterophorus* as indicator species, and (ii) consider both positively and negatively correlated species. The distribution predictions of the indicator species were overlaid using a weighted average by correlation values to construct indicator SDMs, calculated as Equation (3).

(3) $${\mathrm{IRI}}_{\text{Indicator SDMs}}=\frac{{\mathrm{HSI}}_{1}\times r_{1}+{\mathrm{HSI}}_{1}\times r_{1}+\cdots +{\mathrm{HSI}}_{n}\times r_{n}}{\sum_{i=1}^{n}r}$$

${\mathrm{IRI}}_{\text{Indicator SDMs}}$ is the IRI calculated by the indicator SDMs, ${\mathrm{HSI}}_{1\cdots n}$ is the HSI of the selected indicator species, i.e., the EM prediction results, $r_{1\cdots n}$ is the environmental correlation value between the corresponding indicator species and *P. hysterophorus*. To select a more justifiable treatment, the percentage of unsuitable, as well as potential low, moderate, and high invasion risk areas were calculated separately. Finally, taking the distribution of *P. hysterophorus* as a reference, the results of comparison and selection were better, indicating the diffusion trend of *P. hysterophorus* (Figure 2c).

### 2.7 Optimized indicator SDMs construction

Due to the high growth rate of *P. hysterophorus* and its allelopathy, it can rapidly invade new ecosystems and outcompete native species. Thus, indicator SDMs can better identify areas where *P. hysterophorus* has not invaded due to timing or other problems (the Sichuan‐Chongqing region where invasion records have just been found, etc.) or compensate for missing records due to human surveys. In contrast, prediction bias exists for areas that have been successfully invaded, as *P. hysterophorus* is highly susceptible to becoming dominant native species (Ramesh et al., 2017). However, the EM predictions obtained from the training of the currently recorded data can better simulate the areas that have been successfully invaded. To better model the potential invasion risk area of *P. hysterophorus*, we obtained the optimized indicator SDMs by overlaying the prediction results of EM and indicator SDMs for the maximum value, i.e., retaining the maximum value of a single image element of the two prediction results (Figure 2d).

## 3 RESULTS

### 3.1 Indicator species selection

For *P. hysterophorus*, the e‐matrix and r‐matrix can be obtained by constructing the GJAM (Figure S4). Based on the e‐matrix (Figure S3a), we screened two species with positive correlation coefficients, *Cardiospermum halicacabum* (0.933) and *Portulaca oleracea* (0.911), and one species with a negative correlation coefficient, *Crassocephalum crepidioides* (−0.658). According to the MCMC coefficient chains of the selected indicator species (Figure S3), these species were convergent for all seven environmental variables selected. In addition, the three indicator species were widely distributed in the study area (Figure 1b). Therefore, it is rational to use these three indicator species for prediction.

### 3.2 EM accuracy evaluation

**FIGURE 3:**
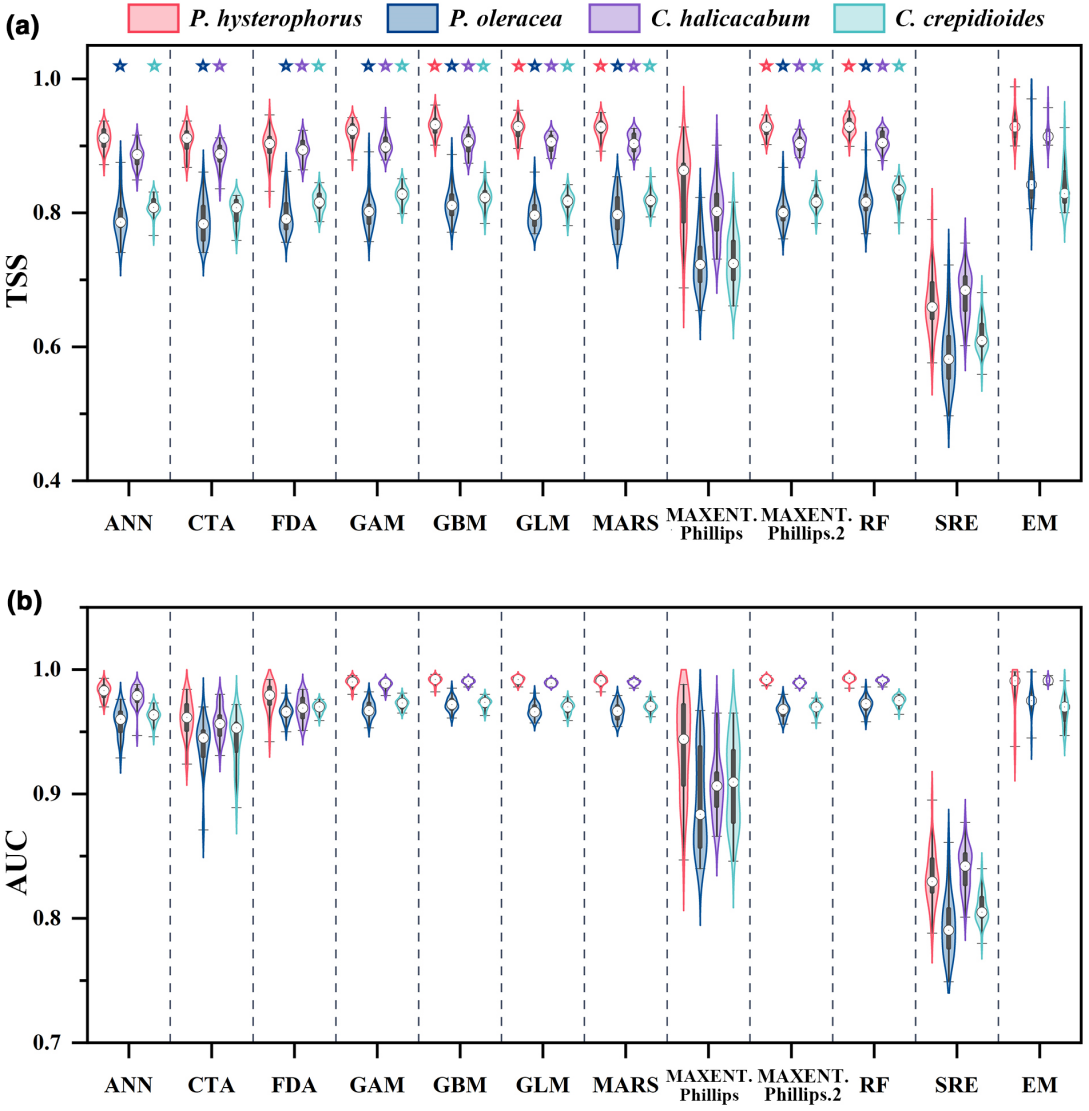
Evaluation indexes of single models and ensemble model (EM) of predicting invasive plant and three indicator species distribution. (a) TSS, (b) AUC. The black box in the middle of each violin plot is the interquartile range. The horizontal lines are the maximum and minimum values, the white circles are the median values. The violin plots show the distribution of the overall data. The pentagrams in figure (a) indicate the single models used to construct the EM. (ANN, artificial neural networks; CTA, classification tree analysis; FDA, flexible discriminant analysis; GAM, generalized additive models; GBM, generalized boosted models; GLM, generalized linear models; MARS, multivariate adaptive regression splines; MAXENT.Phillips and MAXENT.Phillips.2, maximum entropy; RF, random forest; SRE, surface range envelope).

For each species, we calculated the evaluation indexes for 11 kinds of models as well as for the EM (Figure 3). Before constructing the EM, appropriate thresholds were set according to the TSS values of each species (Figure 3a), with 0.9 for *P. hysterophorus* and *C. halicacabum*, 0.8 for *P. oleracea* and *C. crepidioides*. For the four selected species, the EM with the highest TSS as well as AUC values were finally selected (Figure 3b), and the final selected models all had AUC values greater than 0.99 and TSS values greater than 0.92. The single models used to construct the EM for different species were marked with pentagrams (Figure 3a). It can be seen that the GBM, GLM, MARS, MAXENT.Phillips.2, and RF models fit better, while the SRE and MAXENT.Phillips models have a worse fit. Compared to the single model, EM improved the fitting accuracy and reduced the uncertainty.

### 3.3 Comparison with two treatments of the indicator SDMs

**FIGURE 4:**
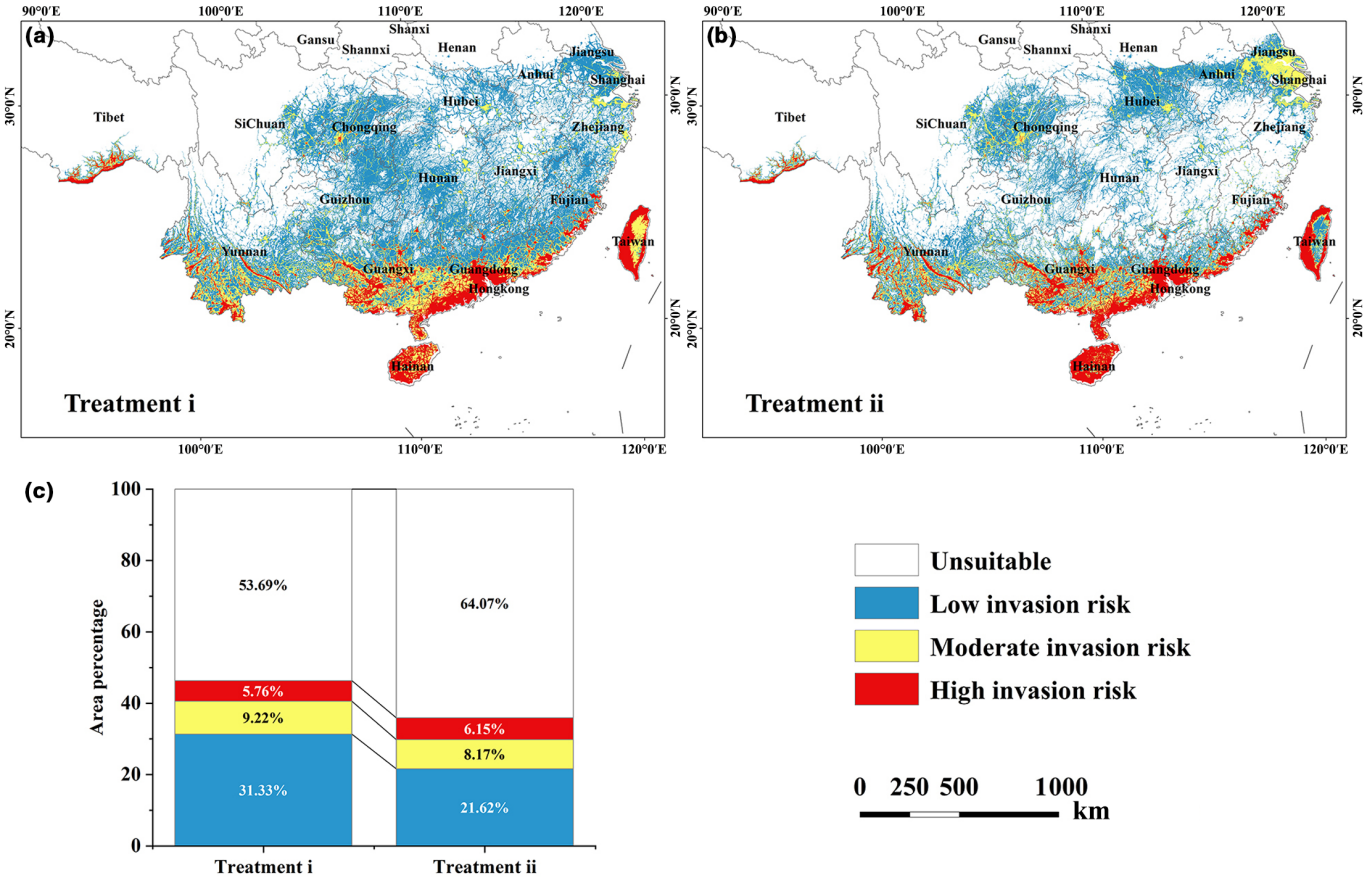
The results of the two treatments of the indicator SDMs. (a) the overlay result of two positively correlated indicator species. (b) the overlay result of two positively correlated and one negatively correlated indicator species. (c) the statistics of the percentage of invasion risk areas with different levels in the results of the two treatments.

Based on the distribution results predicted by EM for the indicator species (Figure S5), indicator SDMs were constructed. The results obtained by indicator SDMs (Figure 4) show that species with only positive correlations (treatment i) predicted more invasion risk areas (46.31%; Figure 4a,c) than species with both positive and negative correlations (treatment ii) (35.93%; Figure 4b,c). For invasion risk levels, with a more pronounced gap in potential low invasion risk areas (9.72%) and a non‐significant gap in potential moderate (1.04%) and high (−0.39%) invasion risk areas (Figure 4c). According to the statistical results, treatment ii predicted fewer risk areas, limiting the species spread trend to a greater extent. As for the distribution, treatment ii showed a small regional concentration, which is more in line with the species' distribution manner. Treatment ii effectively reduced some areas with too high invasion risk in treatment i, such as high altimeters in Taiwan, and strengthened the invasion risk in coastal regions, such as the Shanghai area, etc. Combined with the current distribution of *P. hysterophorus*, treatment ii is more consistent with the reality that long‐distance dispersal is infeasible in the short term. Overall, it is reasonable to use indicator SDMs to predict the potential invasion risk of invasive species, and both positively and negatively correlated species should be taken into account.

### 3.4 Regional variations in prediction results of EM, indicator SDMs, and optimized indicator SDMs

**FIGURE 5:**
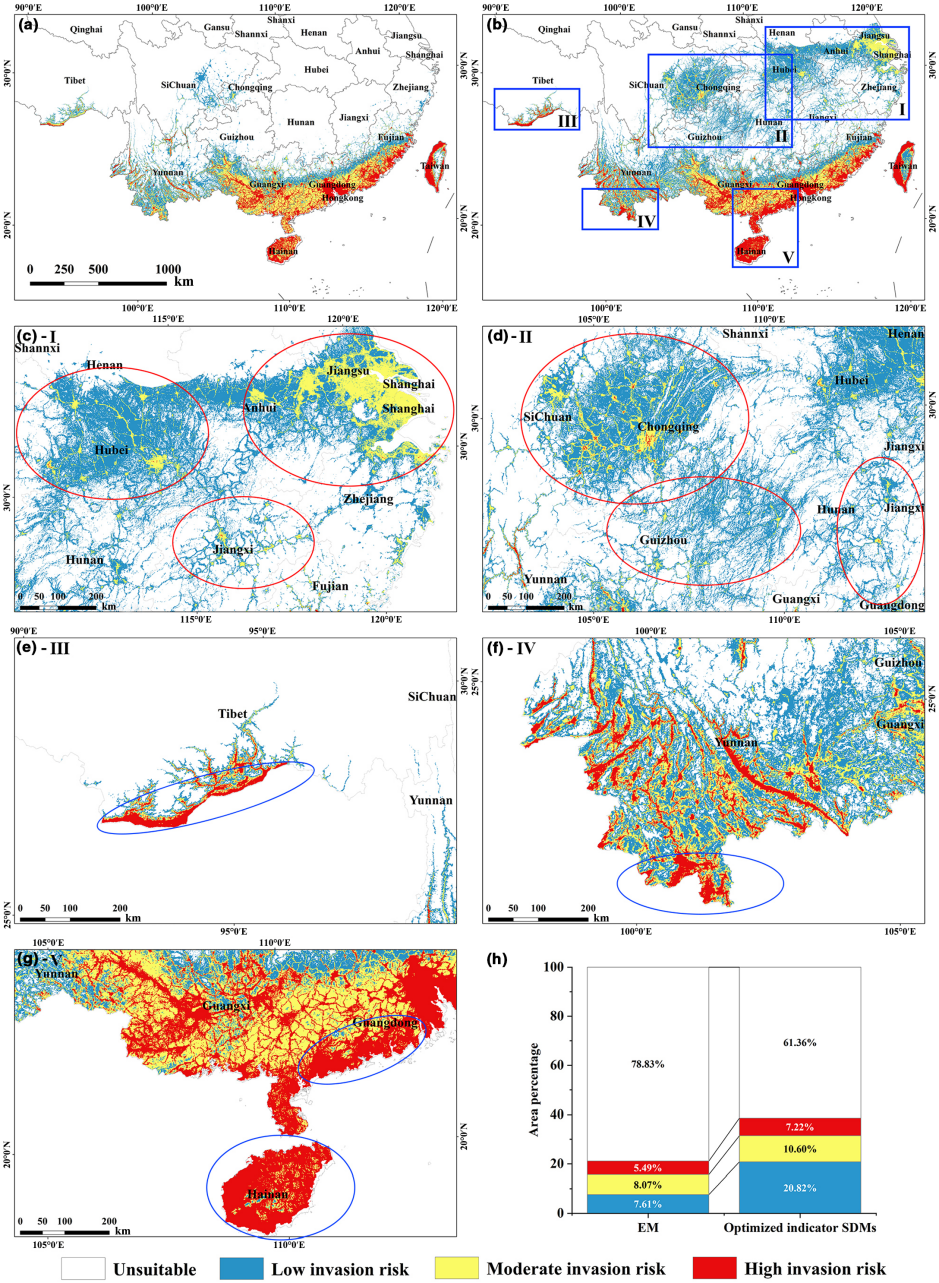
Prediction results of current potential invasion areas of *Parthenium hysterophorus*. (a) predicted by EM, (b) predicted by optimized indicator SDMs. The five blue rectangular boxes in figure (b) are the areas of significant change. (c–g) are enlargements of regions I–V of (b), in the figures, the red ellipses mark the areas where the moderate invasion risk areas have increased significantly, and the blue ellipses are the areas where the high invasion risk areas have increased significantly. (h) the statistics of the percentage of invasion risk areas with different levels in the results of the two models. Where the left is the statistic of (a) and the right is the statistic of (b).

By comparing the prediction results of indicator SDMs (treatment ii; Figure 4b) and EM (Figure 5a), the high invasion risk areas in the coastal region had a higher consistency. However, the EM result depended on the point at which the species occurs (Figure 1c), and predicted more moderate invasion risk areas in the coastal region. In inland zones, invasion risk areas predicted by EM, such as the Sichuan and Chongqing regions, were consistent with the results predicted by the indicator SDMs. Nevertheless, the indicator SDMs had more invasion risk areas, especially in low and moderate invasion risk areas. Further optimization of indicator SDMs is therefore needed.

Compared with the EM (Figure 5a) and indicator SDMs (Figure 4b) prediction results, the prediction of the optimized indicator SDMs included a wider potential invasion risk area while retaining the currently invaded area (Figure 5b). In total, the optimized indicator SDMs predicted 17.47% more potential invasion risk areas compared to EM. Particularly, the potential low invasion risk areas increased significantly by 13.21%, mainly in the interior, and spread in all directions centered on potential moderate and high invasion risk areas (Figure 5b). The potential moderate and high invasion risk areas changed relatively little (Figure 5h). The potential moderate invasion risk areas increased by 2.53%, mainly in the Yangtze River Delta (Figure 5c), with a planar distribution. Potential moderate invasion risk areas also increased in Hubei, Jiangxi (Figure 5c), Chongqing, Sichuan, Guizhou, and Hunan (Figure 5d), showing a point‐like distribution and a linear spread in all directions. For the potential high invasion risk areas, it increased by 1.73%, with more pronounced changes in Tibet (Figure 5e), Yunnan (Figure 5f), Hainan, and Guangdong (Figure 5g). In general, the invasion risk in southeastern coastal and border cities is significantly higher than in other regions, and the overall invasion tends to spread to higher latitudes.

### 3.5 The impact of environment variables on invasion trend

**FIGURE 6:**
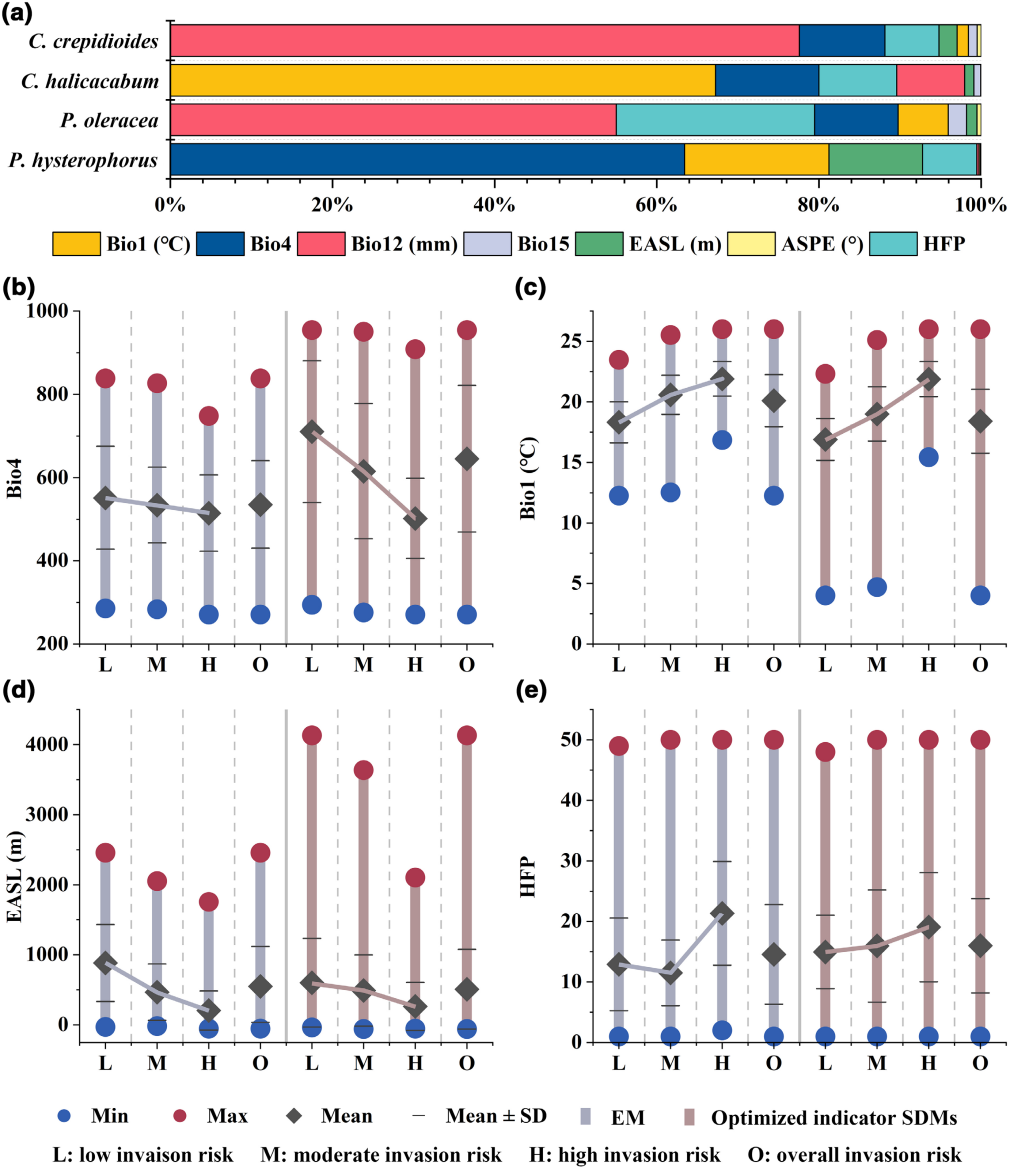
Statistics of the variables used to build the models. (a) the contribution rate of each environmental variable and the cumulative contribution rate of *Parthenium hysterophorus* and its three indicator species based on EM. (b–e) statistics for the four key variables risk areas of *P. hysterophorus*. The four basic statistical indexes for each invasion risk level area and overall invasion risk area (including three levels of low, moderate, and high) are counted.

By building the EM, we could obtain the contribution of each environmental variable for each species (Figure 6a). The environmental variables that had a greater influence on the distribution of *P. hysterophorus* were Bio4 (63.40%), Bio1 (17.81%), EASL (11.54%), and HFP (6.69%). For climatic variables, *P. hysterophorus* was more sensitive to the two selected temperature variables (Bio4 and Bio1), while precipitation variables had less influence. For terrain variables, it was more susceptible to the influence of the EASL variable. For the human activity variable, i.e., HFP, the contribution was small compared to the first three variables.

We calculated threshold ranges, means, and standard deviations for these four key variables (Figure 6b–e: overall). In comparison, the mean values of the four key environmental variables were all changed by the optimized indicator SDMs, and the threshold ranges were expanded for all except the HFP variable. For Bio4, the upper threshold range of 955 (namely, the difference in temperature between the different seasons can reach 9.55°C) was predicted by the optimized indicator SDMs, which was adjusted upwards by 1.16°C. The mean value of Bio4 was 6.45°C, a rise of 1.10°C (Figure 6b). For Bio1, the lower limit of the threshold range was 4°C (namely, the annual mean temperature was 4°C), a decrease of 8.26°C. The mean value of Bio1 was 18.41°C, a decrease of 1.70°C (Figure 6c). By comparing the two temperature variables, *P. hysterophorus* would be more inclusive of seasonal changes in temperature and more tolerant of low temperatures. For EASL, the upper limit of the optimized indicator SDMs reached a maximum of 4133 m, a significant increase of 1674 m. This is probably due to human activities that provide an environment of continuous disturbance over a wide elevation gradient (Dar et al., 2015). The mean value of EASL is 509 m, a drop of 41 m (Figure 6d). For HFP, the mean value increased slightly (1.44) with an index of 15.98 (Figure 6e).

Statistics by each invasion risk level (Figure 6b–e: low, moderate, high) show that both models had the same trend of mean values for different rank changes. That is, for *P. hysterophorus*, regions with smaller seasonal temperature differences, higher annual mean temperatures, lower elevations, and stronger human activities are more vulnerable to invasion. For all key environmental variables, the threshold range for each invasion risk level was expanded by optimized indicator SDMs. For Bio4, the average trend of the optimized indicator SDMs was more pronounced for different classes, where the average of potential low and moderate invasion risk increases significantly and the high invasion risk decreases slightly (Figure 6b). For Bio1, the trend change was not obvious, and the mean value of each rank decreased slightly (Figure 6c). For EASL, the trend change was more stable, with a relatively significant decrease in the mean value of the low and a slight increase in the moderate and high (Figure 6d). For HFP, the trend was flatter, with low and moderate averages increasing and a high invasion risk decreasing.

## 4 DISCUSSION

### 4.1 Feasibility of optimized indicator SDMs prediction

Building appropriate SDMs for invasive species is challenging, especially when applied to species with progressively expanding distributions, where SDMs are severely challenged by underlying assumptions (Gallien et al., 2010; Pack et al., 2022; Pili et al., 2020). Indicator species are selected based on the similarity to the environmental response of invasive species, and these indicator species act as suitable or unsuitable areas for invasive species through positive or negative environmental correlations. Borrowing the indicator species' distribution overcomes the need to rely on specific inter‐species relationships in previous studies (Araújo & Luoto, 2007; Mäkinen & Vanhatalo, 2018; Singer et al., 2018). Moreover, it can partially offset the problem of missing data due to manual sampling density and facilitate large‐scale studies (Singer et al., 2016). For the selection of indicator species, only environmental correlations were considered in this article, but GJAM could yield residual correlations between species (Figure S4b). For species with positive environmental correlations, their residual correlations were also positive (*C. halicacabum*: 0.248; *P. oleracea*: 0.353), indicating a greater overlap of niches between the three species. For species with negative environmental correlations, the residual correlations were small (*C. crepidioides*: 0.080), indicating that this specie has less overlap with the niche of *P. hysterophorus* due to environmental conditions.

The species is experiencing significant range shifts due to intensified human activity and changes in the environment, particularly climate (Elith et al., 2010; Singer et al., 2016). This means that species distribution points no longer reflect a stable relationship between species and their environment. Optimized indicator SDMs integrate current information on the distribution of invasive plants and their indicator species, addressing the problem that the data required in previous studies were costly and could not be generalized to large‐scale studies and most species. In the optimized indicator SDMs prediction result, most of the *P. hysterophorus* occurrence records (Figure 1c) were predicted as potential moderate or high invasion risk areas and spread in all directions along with areas of high human activity (Figure S6). This is consistent with the rapid dispersal mechanism of invasive species (Vila & Ibáñez, 2011).

### 4.2 Optimized indicator SDMs predict wider potential invasion risk areas

The optimized indicator SDMs predicted more potential high invasion risk areas in coastal and border regions compared to EM (Figure 5e–g). Based on the worldwide distribution of *P. hysterophorus* (Figure S7), the origin of this species is similar in latitude to its distribution site in China. Intermittent distribution of North American and East Asian floras is common because of their similar latitudes and geological history, resulting in similar climatic environments (Shou et al., 2014). This, coupled with the fact that coastal and border areas mostly have high import or export logistics and human traffic, provides more convenient conditions for the introduction and settlement of invasive plants (Pan et al., 2015; Tu et al., 2018; Wen & Tu, 2011). Several studies have pointed out that there is a very large amount of suitable habitat for *P. hysterophorus* in eastern China, but it has not yet been recorded (Maharjan et al., 2020; Mainali et al., 2015). And the optimized indicator SDMs can well predict the potential suitable habitats in eastern China.

Alternatively, the optimized indicator SDMs predicted broader invasion risk areas in the interior (Figure 5b). In general, the key processes of biological invasion include establishment, dispersal, and becoming influential or even dominant members of a community (Blackburn et al., 2011). Invasive plants can only reach high density and large‐scale spatial distributions through dispersal behavior, creating a true invasion (Ou & Lu, 2006). *P. hysterophorus* was first discovered in Yunnan, a region with high topography. During the seed maturation season (summer and autumn), southwestern monsoons prevail and are easily dispersed by wind to the northeast. For invasive plants (e.g. *P. hysterophorus*) that are widely distributed near rivers (Yangtze River) and coasts, the water flow will promote the spread of them inland to high latitudes as well as along the coast, such as the Yangtze River pathway area (Jiang, 2018), parts of Jiangxi, and Yangtze River Delta, etc. (Figure 5c). Moreover, in coastal areas, the thermal properties difference between the land and the sea in creates summer winds that blow from the southeast to the northwest, driving the spread of invasive plants. Together with the convenient transportation conditions, invasive plants can further spread in the inland areas.

### 4.3 Optimized indicator SDMs predict a broader niche breadth

Most invasive plants have changed their niche in introduced ranges due to the lack of inter‐restricted species or dispersal limits in the invaded site (Atwater et al., 2018; Broennimann et al., 2007; Pili et al., 2020). In this article, we calculated the contribution of environmental variables (Figure 6a) and obtained four key environmental variables (Bio4, Bio1, EASL, and HFP) influencing the distribution of *P. hysterophorus*. Compared to EM, the range of thresholds for all key variables except HFP was expanded for the optimized indicator SDMs, but the mean values did not change significantly (Figure 6b–e). This is mainly because human activities cause its diffusion, thus disturbing its environmental variables thresholds (Cardador & Blackburn, 2020). Bio4 variable showed the most significant change (Figure 6b), and it is also the variable that most affects the distribution of *P. hysterophorus* (Figure 6a). This change indicates that *P. hysterophorus* will be more inclusive of seasonal temperature differences, further indicating its ability to disperse northward.

### 4.4 Optimized indicator SDMs highlight the impact of human activities on the distribution of invasive plants

Based on the optimized indicator SDMs prediction result, the distribution of *P. hysterophorus* showed a tendency to spread in a punctiform manner and along with areas of high human activity (Figure 5b; Figure S6a). Invasive plants are subject to human activities that allow them to cross biogeographic barriers and enhancing genetic diversity in new populations, thereby becoming established in one location (Bertelsmeier & Keller, 2018; Richardson, 2008; Wilson et al., 2009). Habitat fragmentation due to anthropogenic disturbance allows invasive plants to spread much faster and wider than natural and animal dispersal (Vila & Ibáñez, 2011; Yan et al., 2012). Overlaying the road network (https://www.openstreetmap.org/) and HFP variable (Figure S6b) reveals that areas of high human activity have higher road densities. Roads, as corridors that facilitate the spread of invasive plants, also have a strong influence on the distribution and abundance patterns of invasive plants. Roads and human activity cause great anthropogenic disturbance to the surrounding natural communities and lead to habitat fragmentation (Dar et al., 2015). The optimized indicator SDMs predicted more potential invasion risk areas in areas of high human activity, which is consistent with the dispersal mechanism of invasive species.

### 4.5 Optimized indicator SDMs facilitate the management of invasive species

By comparing the current distribution of invasive plants (SDMs prediction) with their potential invasion risk areas (optimized indicators SDMs prediction), invasive sites can be divided and management measures can be proposed for the different divisions. For example, in areas that are newly invaded but not fully spread, invasive plants should be destroyed before flowering or fruiting to prevent seed production, and then sown with desirable crops. Monitoring should be intensified in areas with strong human activity (roadsides, next to buildings, etc.) and open areas. For areas that have not been invaded but are at potential invasion risk, quarantine should be strengthened to prevent in advance. For areas that have already been invaded, invasive plants can be controlled with chemical herbicides. For large areas that are invaded, biological control can be used for economic and environmental reasons. Overall, To prevent and control invasive plants, it is imperative to focus on the spread and dispersal due to anthropogenic causes (Sayit et al., 2019) and reduce their spread to new areas (Bajwa et al., 2016).

## 5 CONCLUSIONS

We used the GJAM model in JSDMs to select species highly related to *P. hysterophorus* as indicator species. Then, we simulated the distribution of *P. hysterophorus* and the indicator species with EM. Finally, we combined the distribution information of the indicator species with that of *P. hysterophorus* to construct optimized indicator SDMs to predict the potential invasion risk of *P. hysterophorus*. The results showed that the traditional SDMs relied on the current species distribution points and had limitations for invasive plants with a spreading range. However, the optimized indicator SDMs are not only effective in predicting a wider distribution range but also in indicating the contribution of human activities to the spread of invasive plants. Based on such predictions, rational management of *P. hysterophorus* can be carried out earlier.

## AUTHOR CONTRIBUTIONS

**Jiamin Liu:** Conceptualization (equal); data curation (lead); formal analysis (lead); methodology (lead); software (lead); writing – original draft (lead); writing – review and editing (equal). **Haiyan Wei:** Conceptualization (supporting); funding acquisition (equal); supervision (supporting); writing – review and editing (supporting). **Jiaying Zheng:** Data curation (supporting); formal analysis (supporting); software (supporting). **Ruidun Chen:** Data curation (supporting); formal analysis (supporting). **Lukun Wang:** Data curation (supporting); formal analysis (supporting). **Fan Jiang:** Data curation (supporting); formal analysis (supporting). **Wei Gu:** Conceptualization (equal); funding acquisition (equal); supervision (lead); writing – review and editing (equal).

## FUNDING INFORMATION

The Natural Science Basic Research Program of Shaanxi Province, Grant/Award Number: 2020JM‐277; the National Natural Science Foundation of China, Grant/Award Number: 31070293; the Research and Development Program of Science and Technology of Shaanxi Province, Grant/Award Number: 2014K‐01‐02.

## CONFLICT OF INTEREST STATEMENT

The authors declare no conflict of interest.

## Supporting information


Appendix S1

## Data Availability

All environmental variables used in the manuscript are already publicly accessible, and we have provided the download address in the manuscript. Species occurrence data used in this manuscript are uploaded in Dyard (https://doi.org/10.5061/dryad.kprr4xh7c).
