# Supplementary material for: Constructing indicator species distribution models to study the potential invasion risk of invasive plants: A case of the invasion of Parthenium hysterophorus in China
Source: Ecol Evol. 2023 Nov 1;13(11):e10672. doi: 10.1002/ece3.10672 (PMC10618719; doi:10.1002/ece3.10672)
Supplement: Supplementary file 1 — Appendix S1 [file ECE3-13-e10672-s001.docx]

Table S1. GBIF download DOIs and associated information

| **Species** | **GBIF download information (DOI)** |
| --- | --- |
| Taiwan all species | DOI: https://doi.org/10.15468/dl.dsu3dw  Creation Date: 11:03:21 15 July 2021  Records included: 316197 records from 75 published datasets |
| *Parthenium hysterophorus* | DOI: https://doi.org/10.15468/dl.kn2cz2  Creation Date: 13:01:00 13 December 2021  Records included: 35 records from 9 published datasets  DOI: https://doi.org/10.15468/dl.2nfvnh (World)  Creation Date: 06:12:59 21 March 2022  Records included: 10263 records from 198 published datasets |
| *Cardiospermum halicacabum* | DOI: https://doi.org/10.15468/dl.qusyxf  Creation Date: 05:37:24 16 December 2021  Records included: 133 records from 13 published datasets |
| *Portulaca oleracea* | DOI: https://doi.org/10.15468/dl.87eam3  Creation Date: 13:39:31 20 December 2021  Records included: 289 records from 17 published datasets |
| *Crassocephalum crepidioides* | DOI: https://doi.org/10.15468/dl.p2ta9t  Creation Date: 15:45:54 28 February 2022  Records included: 468 records from 25 published datasets |

Table S2. Data sources and references for environmental variables.

| Environment variables | Sources | Description | Reference/software* |
| --- | --- | --- | --- |
| Climate | WorldClim 2.1: http://www.worldclim.org | Bio1 ~ Bio19 | Fick & Hijmans, 2017 |
|  | ENVIREM dataset: https://envirem.github.io/ | Annual potential evapotranspiration (annual PET) and climatic moisture index (CMI) | Title & Bemmels, 2018 |
| Terrain | Extracted from the digital elevation data (DEM) (http://www.resdc.cn/) of ArcGIS 10.7 | Slope gradient (SLOP), elevation above sea level (EASL), and slope direction (ASPE) | ESRI, 2019 |
|  | ENVIREM dataset: https://envirem.github.io/ | Terrain roughness index (TRI) and SAGA-GIS topographic wetness index (TWI) | Title & Bemmels, 2018 |
| Human activity | The Socioeconomic Data and Applications (https://sedac.ciesin.Columbia.edu/) | Human footprint dataset (HFP) | Venter et al., 2016 |

***** ESRI, 2019. ArcGIS Desktop: Release 10.7.

Fick, S. E., & Hijmans, R. J. (2017). WorldClim 2: new 1-km spatial resolution climate surfaces for global land areas. International Journal of Climatology, *37*, 4302–4315. https://doi.org/10.1002/joc.5086

Title, P. O., & Bemmels, J. B. (2018). ENVIREM: an expanded set of bioclimatic and topographic variables increases flexibility and improves performance of ecological niche modeling. *Ecography*, *41*, 291–307. https://doi.org/10.1111/ecog.02880

Venter, O., Sanderson, E. W., Magrach, A., Allan, J. R., Beher, J., Jones, K. R., Possingham, H. P., Laurance, W. F., Wood, P., Fekete, B. M., Levy, M. A., & Watson, J. E. M. (2016). Sixteen years of change in the global terrestrial human footprint and implications for biodiversity conservation. *Nature Communications*, *7*, 1–11. https://doi.org/10.1038/ncomms12558

Table S3. Detailed species descriptions of *P. hysterophorus* and its 27 community members.

| **Species name*** | **Order** | **Family** | **Life form** | **Habitat** |
| --- | --- | --- | --- | --- |
| *Bidens pilosa* | Asterales | Asteraceae | Annual herbs | Near villages, roadside, or wasteland. |
| *Broussonetia papyrifera* | Rosales | Moraceae | Arbor | Near the village of wasteland, pastoral, or ditch. |
| ***Cardiospermum halicacabum*** | Sapindales | Sapindaceae | Herbaceous climbing vine | Fields, thickets, roadside, or forest margins. |
| *Chromolaena odorata* | Asterales | Asteraceae | Perennial herbs | Low-elevation hills, thickets, savannas, dry land, forest damage, wasteland, roadside, residential, or fields. |
| ***Crassocephalum crepidioides*** | Asterales | Asteraceae | Erect herbs | Hillside roadside, waterside, or thicket. |
| *Cyperus iria* | Poales | Cyperaceae | Annual herbs | Field, hillside, or roadside damp place. |
| *Digitaria sanguinalis* | Poales | Poaceae | Annual herbs | Roadside or fields. |
| *Eleusine indica* | Poales | Poaceae | Annual herbs | Barren places or near roads. |
| *Emilia sonchifolia* | Asterales | Asteraceae | Annual herbs | Hillside wasteland, ridge, or roadside. |
| *Ipomoea cairica* | Solanales | Convolvulaceae | Perennial twining herbs | Flat or mountain roadside thickets, or sunny place. |
| *Ipomoea obscura* | Solanales | Convolvulaceae | Twining herbs | Deserts, sand, by the sea, open woods, or thickets. |
| *Ipomoea triloba* | Solanales | Convolvulaceae | Herbs | Hilly roadsides, grassy fields, or fields. |
| *Lantana camara* | Lamiales | Verbenaceae | Shrubs or creeping shrubs | Beach or open areas. |
| *Leucaena leucocephala* | Fabales | Fabaceae | Shrubs or small trees | Lowland or open forest. |
| *Mimosa pudica* | Fabales | Fabaceae | Loose, sub-shrubby herbs | Wilderness or bush. |
| *Oxalis corniculata* | Oxalidales | Oxalidaceae | Herbs | Hillside grass pool, river valley along the side of the road, the edge of the field, wasteland or forest damp place. |
| *Paederia foetida* | Gentianales | Rubiaceae | Vines shrubs | Sparse forests at low altitudes. |
| ***Parthenium hysterophorus*** | Asterales | Asteraceae | Annual herbs | Open land, roadside, river, or slope. |
| *Passiflora foetida* | Malpighiales | Passifloraceae | Herbaceous vine | At an altitude of 120-500 meters grassy slope roadside. |
| ***Portulaca oleracea*** | Caryophyllales | Portulacaceae | Annual herbs | Vegetable gardens, farmland, roadside, or common weeds in the field. |
| *Sida rhombifolia* | Malvales | Malvaceae | Erect subshrub | Hillsides, thickets, open fields, or along the banks of valleys. |
| *Solanum americanum* | Solanales | Solanaceae | Delicate herbs | Streamside, dense forest dank place, or forest side wasteland. |
| *Solanum nigrum* | Solanales | Solanaceae | Annual herbs | The edge of a field, a road, or a wasteland. |
| *Stephania japonica* | Ranunculales | Menispermaceae | Slightly woody vines | The edge of a village or wilderness thicket. |
| *Symphyotrichum subulatum* | Asterales | Asteraceae | Annual herbs | Thickets, grassy slopes, ditches, roadside, or wasteland. |
| *Synedrella nodiflora* | Asterales | Asteraceae | Annual herbs | Wilderness, arable land, roadside or house side, strong fertility. |
| *Tridax procumbens* | Asterales | Asteraceae | Perennial paving herbs | Low altitude wilderness, wasteland, slope, or roadside sun. |
| *Youngia japonica* | Asterales | Asteraceae | Perennial herbs | Hillside, valley and gully forest edge, forest, forest grassland, wetland, river marsh, field, or wasteland. |

* The invasive plant in this study is marked in red font, and its indicator species are in orange (positive) and green (negative) font.

Table S4. Details of the models included in the "biomod2" package

| Models | Introduction | Reference* |
| --- | --- | --- |
| artificial neural networks (ANN) | It is based on a non-linear mapping structure of human brain functions and has proven to be a versatile and highly flexible approximator of functions for any data. | Lek & Guégan, 1999 |
| classification tree analysis (CTA) | It uses recursive partitioning to split the data into increasingly smaller, homogenous subsets until termination is reached. | Vayssières et al., 2000 |
| flexible discriminant analysis (FDA) | It is a general methodology that aims at providing tools for multigroup non-linear classification. | Hastie et al., 1994 |
| generalized additive models (GAM) | It is a data-driven, non-parametric extension of the generalized linear model, which can be fitted to non-linear relationships. | Guisan et al., 2002 |
| generalized boosted models (GBM) | It is designed to fit many simple models whose predictions are then combined to give more robust estimates of the relationship between species distribution and a set of environmental variables | Elith et al., 2008 |
| generalized linear models (GLM) | It is a generalization of general linear models, preferable for a nonlinear yet simple relationship between species and environment variables. | Guisan et al., 2002 |
| multivariate adaptive regression splines (MARS) | It is a non-parametric regression method that builds multiple linear regression models across the range of predictor values. | Friedman, 1991 |
| maximum entropy (Maxent, the "biomod2" package contains two types: "MAXENT.Phillips", "MAXENT.Phillips.2". | The rationale of Maxent is to estimate a target probability distribution by finding the probability distribution of maximum entropy, subject to a set of constraints that represent the species distribution. | Phillips et al., 2006 |
| random forest (RF) | It is an ensemble classifier that consists of many decision trees and is not sensitive to the problem of multicollinearity. | Breiman, 2001 |
| surface range envelope (SRE) | Surface Range Envelope is an envelope-style method similar to Bioclim. It is a presence-only model that uses the environmental conditions of locations of occurrence data to profile the environments where a species can be found. | Busby, 1991 |

* Breiman, L. (2001). Random forests. *Machine Learning*, *45*, 5–32.

Busby, J. R. (1991). BIOCLIM-a bioclimate analysis and prediction system. *Plant Protection Quarterly*, *6*, 8–9.

Elith, J., Leathwick, J. R., & Hastie, T. (2008). A working guide to boosted regression trees. *Journal of Animal Ecology*, *77*, 802–813. https://doi.org/10.1111/j.1365-2656.2008.01390.x

Friedman, J. H. (1991). Multivariate adaptive regression splines. *The Annals of Statistics*, *19*, 1–67.

Guisan, A., Edwards Jr, T. C., & Hastie, T. (2002). Generalized linear and generalized additive models in studies of species distributions: setting the scene. *Ecological Modelling*, *157*, 89–100. https://doi.org/10.1016/S0304-3800(02)00204-1

Hastie, T., Tibshirani, R., & Buja, A. (1994). Flexible discriminant analysis by optimal scoring. *Journal of the American Statistical Association*, *89*, 1255–1270. https://doi.org/10.1080/01621459.1994.10476866

Lek, S., & Guégan, J.-F. (1999). Artificial neural networks as a tool in ecological modelling, an introduction. *Ecological Modelling*, *120*, 65–73. https://doi.org/10.1016/S0304-3800(99)00092-7

Phillips, S. J., Anderson, R. P., & Schapire, R. E. (2006). Maximum entropy modeling of species geographic distributions. *Ecological Modelling*, *190*, 231–259. https://doi.org/10.1016/j.ecolmodel.2005.03.026

Vayssières, M. P., Plant, R. E., & Allen‐Diaz, B. H. (2000). Classification trees: An alternative non‐parametric approach for predicting species distributions. *Journal of Vegetation Science*, *11*, 679–694. https://doi.org/10.2307/3236575


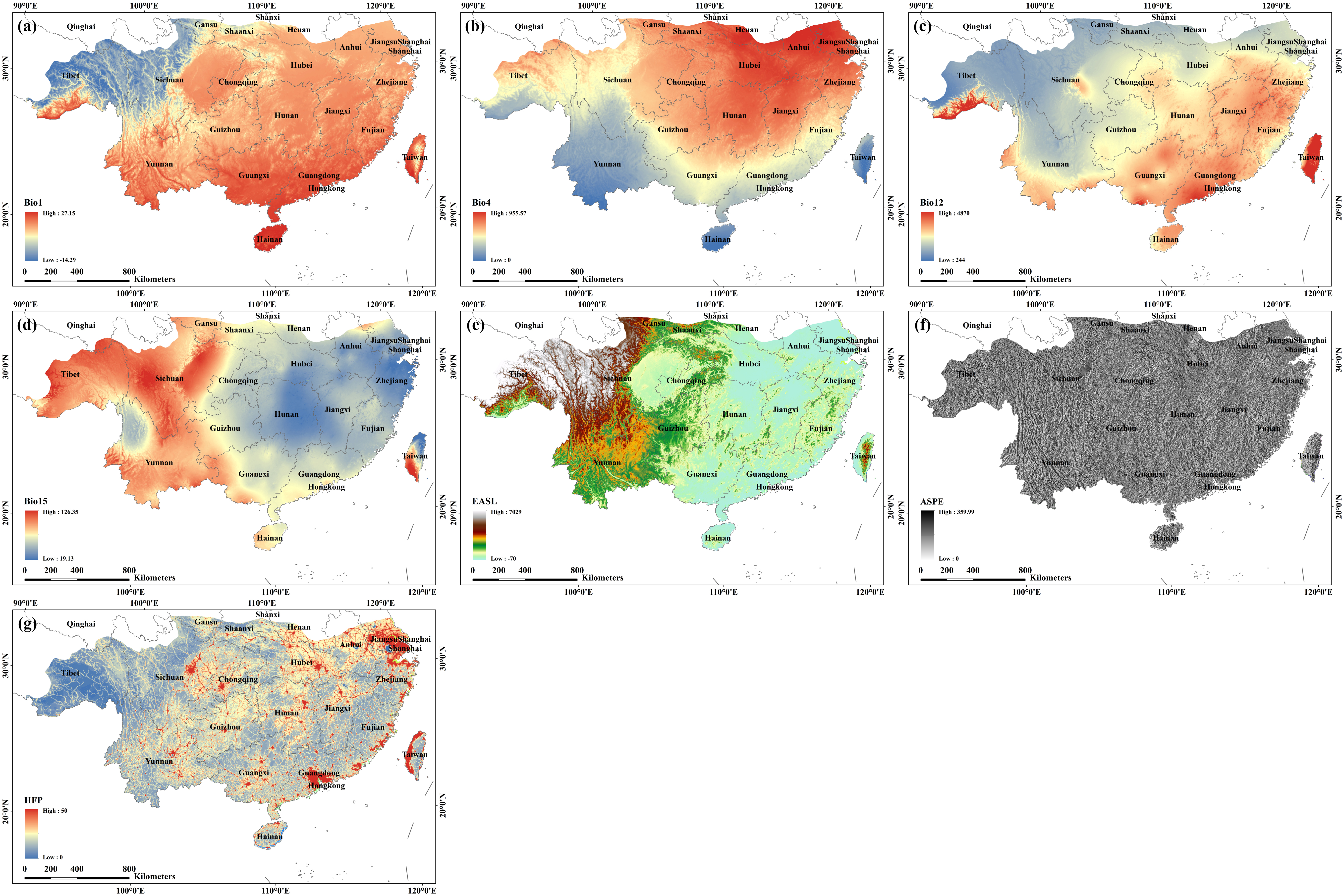


Figure S1. Environment variables after the selection. (a) Bio1, (b) Bio4, (c) Bio12, (d) Bio15, (e) EASL, (f) ASPE, and (g) HFP.


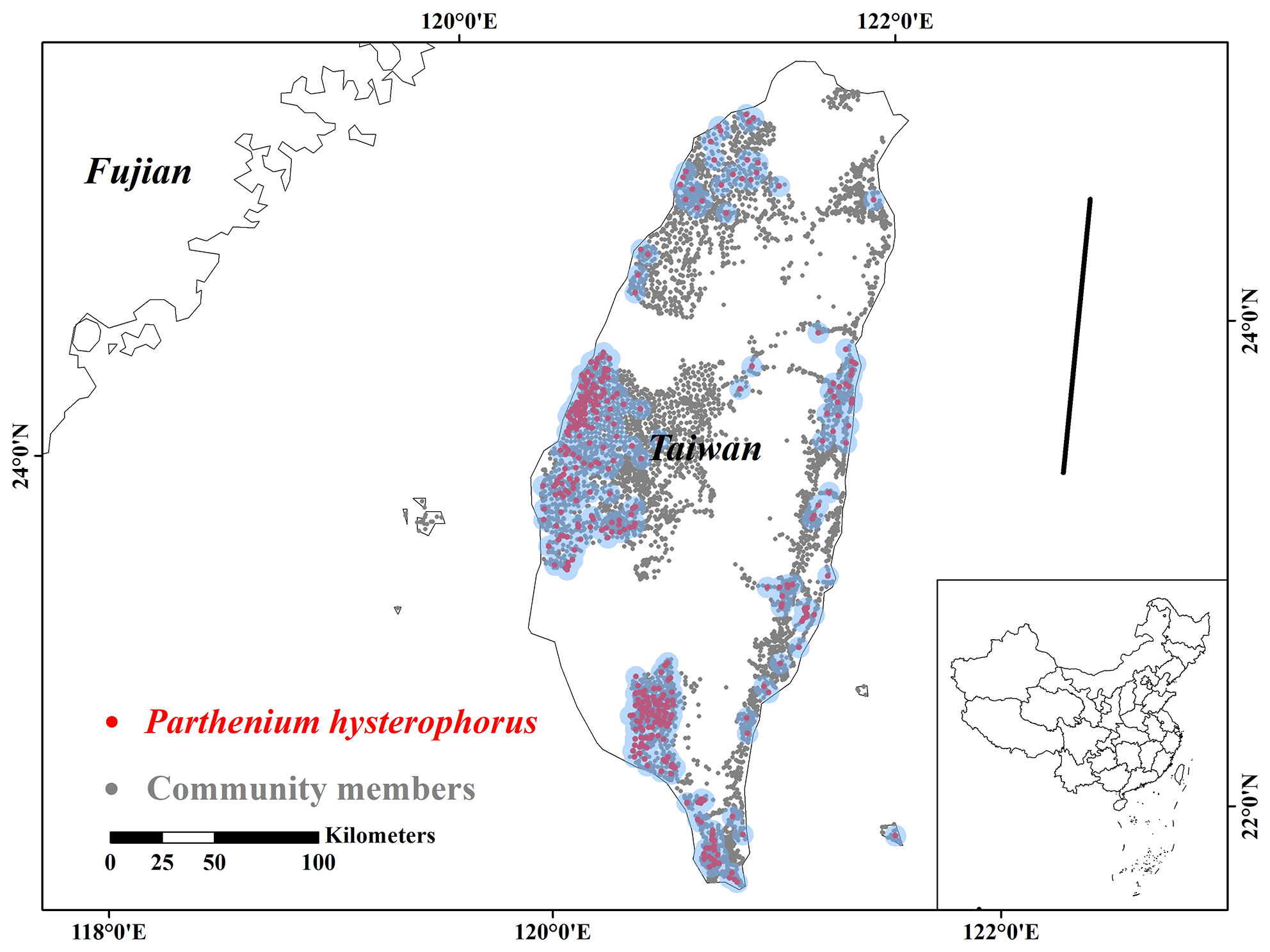


Figure S2. The species occurrence data used in GJAM. Red points are the distribution spots of *P.* *hysterophorus.* The gray dots are the locations of the 27 community members. The pale blue circle of each *P.* *hysterophorus* distribution point is the 5 km buffer zone of that point.


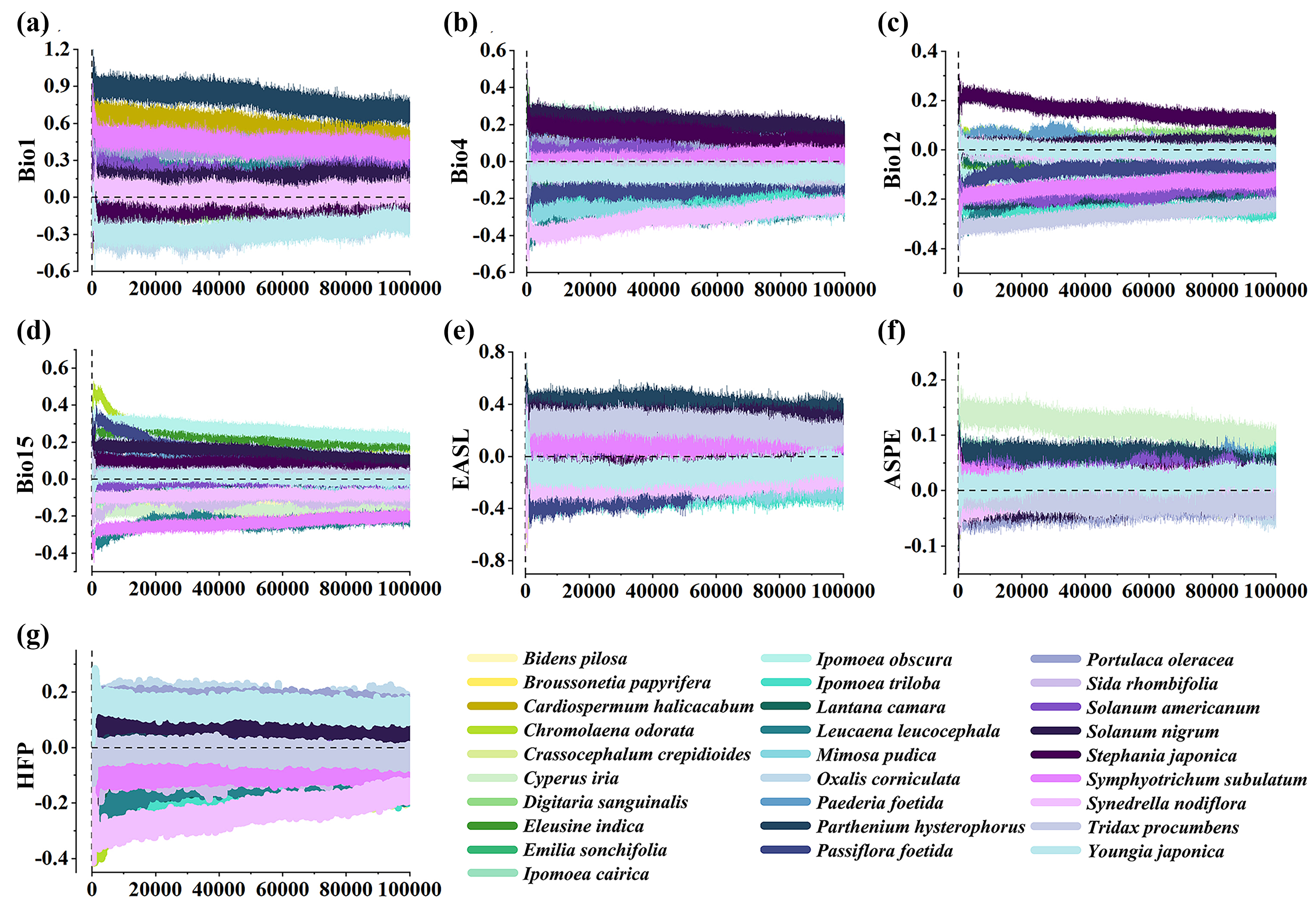


Figure S3. MCMC coefficient chains for 27 community members and *P. hysterophorus* in GJAM*.* The convergence of MCMC chains can indicate the reasonableness of the choice of predictor variables. (a) Bio1, (b) Bio4, (c) Bio12, (d) Bio15, (e) EASL, (f) ASPE, and (g) HFP.


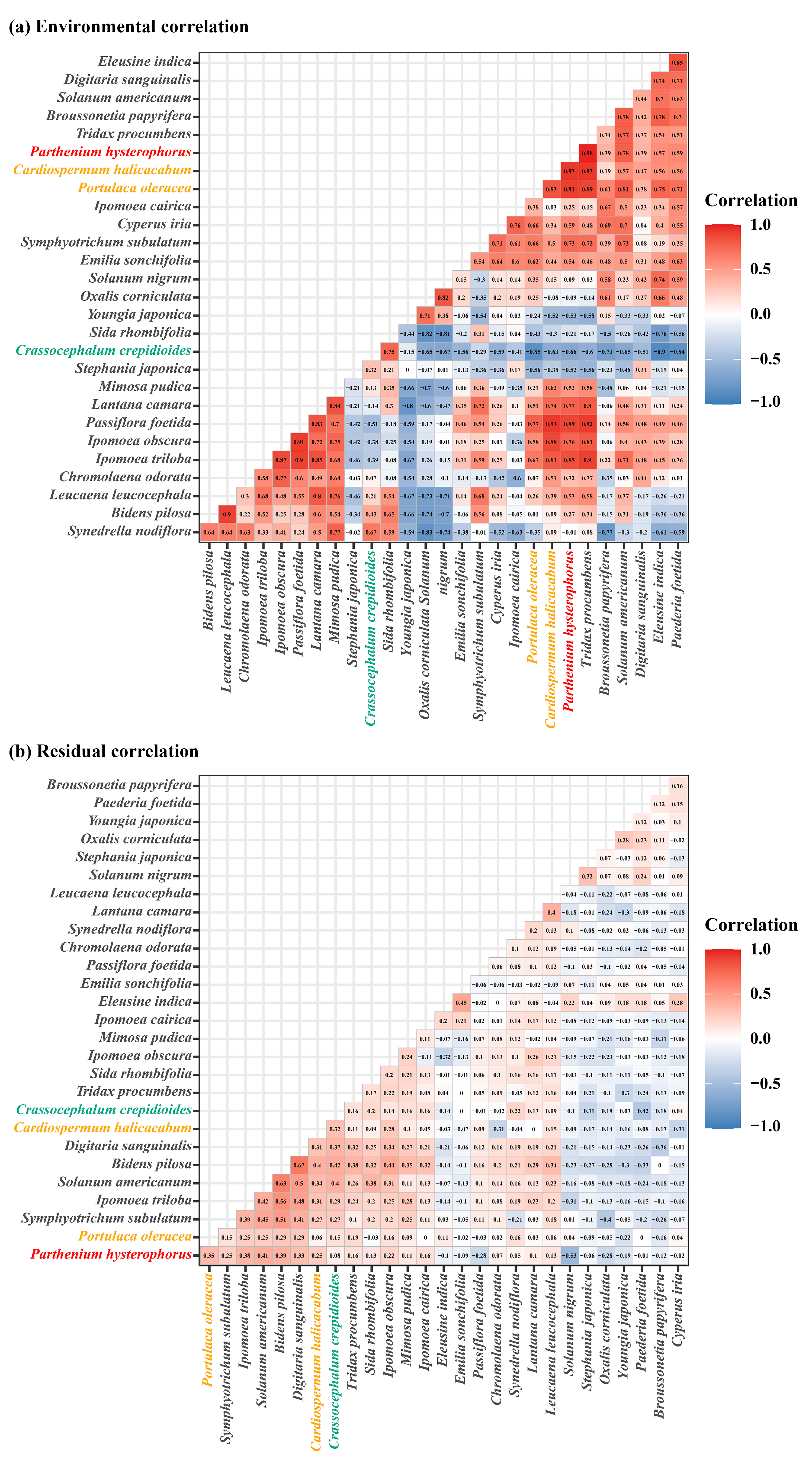


Figure S4. The correlation matrix of the GJAM result. (a) e-matrix, (b) r-matrix. The red font represents the invasive plant *P. hysterophorus*. The yellow font represents those with a positive correlation with *P. hysterophorus*, and the green font represents the specie with a negative correlation.


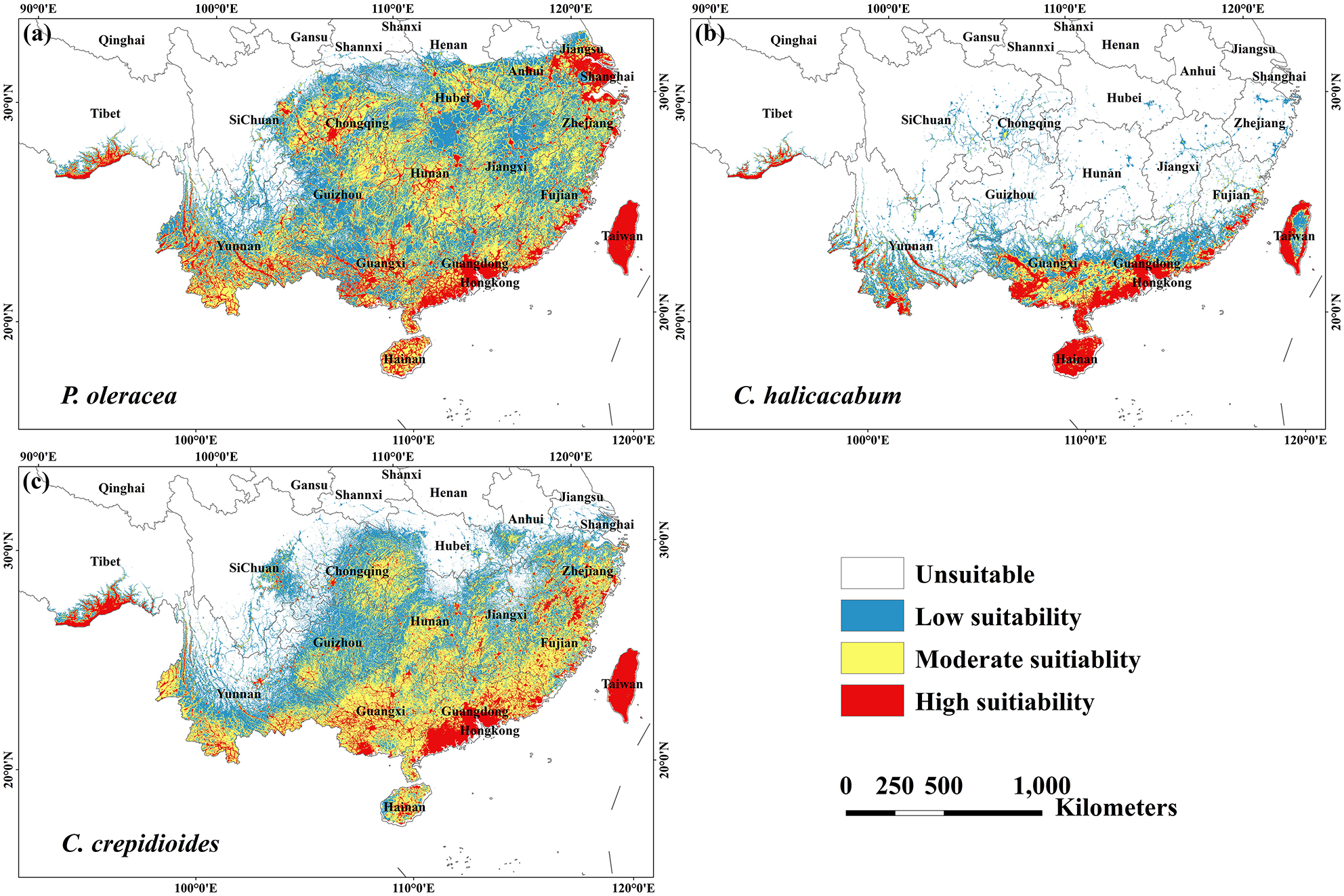
Figure S5. The predicted suitable distribution of the three indicator species by EM in the study area. (a) *P. oleracea*, (b) *C. halicacabum*, (c) *C. crepidioides*.


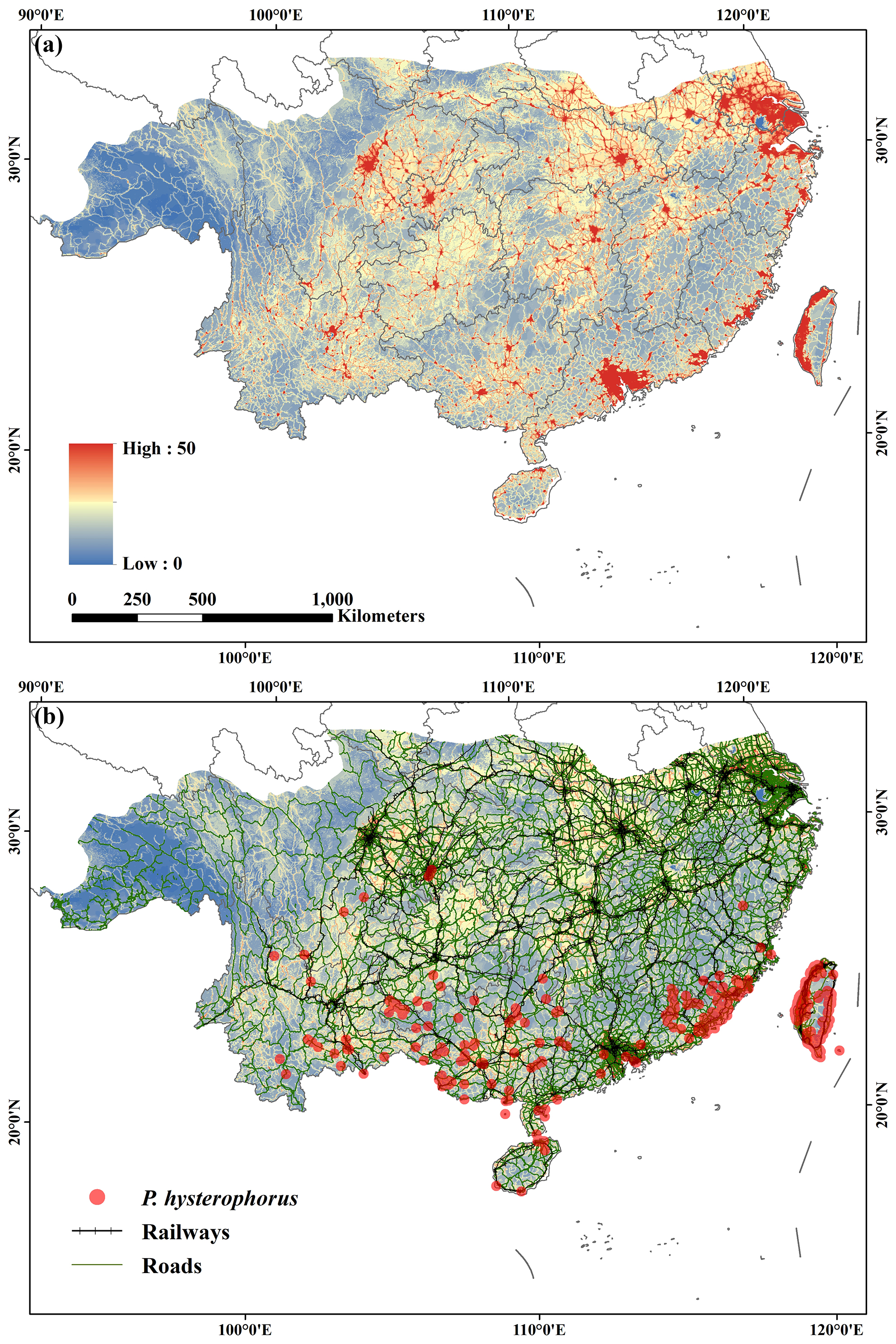


Figure S6. Overlay of HFP variables and road network with *P. hysterophorus* distribution. (a) human footprint variable in the study area. (b) the road network in the study area. The black lines are railroads, and the green lines refer to highways, arterial roads, and major urban roads.


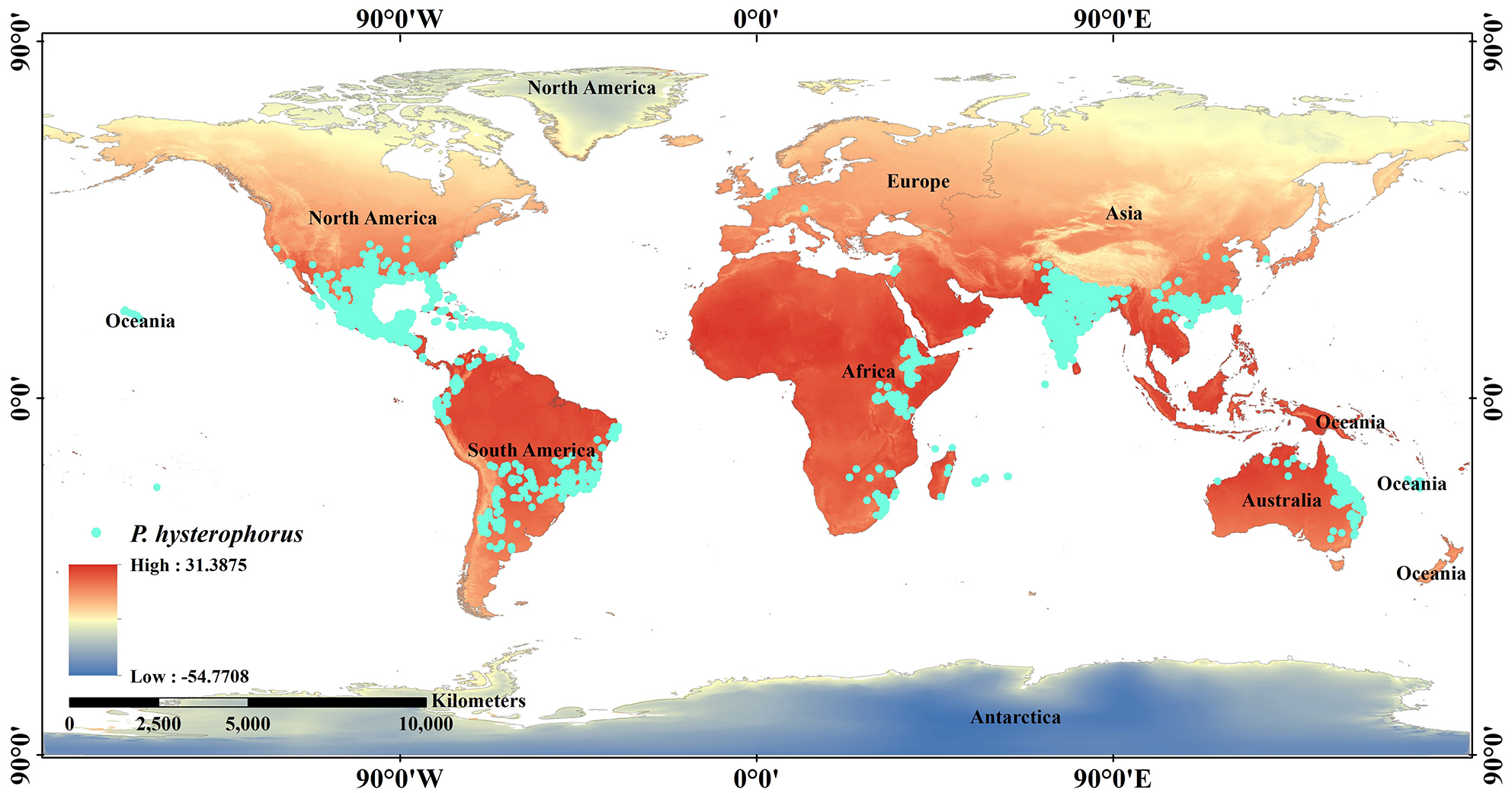


Figure S7. The world species occurrence of *P. hysterophorus*. The base map is climate variable bio1, cyan points are specie occurrence points.
